# Characterization of human aquaporin ion channels in a yeast expression system as a tool for novel ion channel discovery

**DOI:** 10.1042/BSR20240542

**Published:** 2024-08-28

**Authors:** Saeed Nourmohammadi, Sam W. Henderson, Sunita A. Ramesh, Andrea J. Yool

**Affiliations:** 1 School of Biomedicine, Faculty of Health and Medical Sciences, and the Institute for Photonics and Advanced Sensing, University of Adelaide, Adelaide, SA 5005, Australia; 2 Biological Sciences, College of Science and Engineering, Flinders University, Bedford Park, SA 5042, Australia

**Keywords:** aquaporins, drug discovery and design, high-throughput screening, ion channels, Saccharomyces cerevisiae

## Abstract

Aquaporin (AQP) channels found in all domains of life are transmembrane proteins which mediate passive transport of water, glycerol, signaling molecules, metabolites, and charged solutes. Discovery of new classes of ion-conducting AQP channels has been slow, likely reflecting time- and labor-intensive methods required for traditional electrophysiology. Work here defines a sensitive mass-throughput system for detecting AQP ion channels, identified by rescue of cell growth in the K^+^-transport-defective yeast strain CY162 following genetic complementation with heterologously expressed cation-permeable channels, using the well characterized human AQP1 channel for proof of concept. Results showed AQP1 conferred transmembrane permeability to cations which rescued survival in CY162 yeast. Comprehensive testing showed that growth response properties fully recapitulated AQP1 pharmacological agonist and antagonist profiles for activation, inhibition, dose-dependence, and structure–function relationships, demonstrating validity of the yeast screening tool for AQP channel identification and drug discovery efforts. This method also provided new information on divalent cation blockers of AQP1, pH sensitivity of antagonists, and ion permeability of human AQP6. Site-directed mutagenesis of AQP1 channel regulatory domains confirmed that yeast growth rescue was mediated by the introduced channels. Optical monitoring with a lithium-sensitive photoswitchable probe in living cells independently demonstrated monovalent cation permeability of AQP1 channels in yeast plasma membrane. Ion channel properties of AQP1 expressed in yeast were consistent with those of AQP1 expressed in *Xenopus laevis* oocyte and K^+^-transport defective *Escherichia coli*. Outcomes here establish a powerful new approach for efficient screening of phylogenetically diverse AQPs for yet untested functions as cation channels.

## Introduction

Following the first discovery of an aquaporin (AQP) with ion channel functionality [1], at least ten additional subtypes of AQPs have been discovered to be cation or anion channels to date [2,3]. With thousands of orthologs in the genomic database, it is likely many other AQP classes across phyla also have combined water and ion channel functions that remain unknown. Gated AQP1 monovalent cation permeability contributes to diverse processes including regulation of cell volume, fluid secretion and absorption, motility, and responses to environmental stressors [3–6], with more roles likely to be discovered not just for AQP1 but for many other classes of AQP channels. A novel high-throughput screening method is needed to accelerate scientific progress in this field by providing an unbiased tool for efficient discovery and characterization of ion channel properties across diverse AQP classes, the vast majority of which remain untested because possible activating signals are unknown, and pharmacological tools needed for modifying functional roles have not been identified.

The *Saccharomyces cerevisiae* CY162 line, genetically deficient in K^+^ uptake [91], has proven useful for assessing functions of heterologously expressed cation channels and transporters detected by the rescue of yeast cell survival via functional complementation, with cell growth serving as a proxy measure for K^+^ ion flux [7–10]. Work here tested the idea that CY162 mass screening is a sensitive method for identifying aquaporin cation channels. Efficient screening of candidate activation pathways would resolve a major barrier to progress in the field. In a few studies, *S. cerevisiae* has been used to investigate aspects of ion permeation associated with AQPs. Byrt and colleagues demonstrated cytosolic accumulation of Na^+^ in yeast cells expressing *Arabidopsis thaliana* AtPIP2;1 [11], and showed C-terminal phosphorylation of AtPIP2;1 controlled ion permeability in a Na^+^-transport deficient yeast strain [12]. With the K^+^- and Na^+^-sensitive yeast strains CY162 and MA5, Beitz and colleagues showed that combined point mutations in the intrasubunit pore selectivity filters of rat AQP1 reduced selectivity for water, enabling high proton permeation as well as transit of other less permeable monovalent cations [13].

The aim of the present study was to evaluate the suitability of the CY162 expression system as a platform for high-throughput screening of diverse classes of AQPs, using the most studied aquaporin ion channel, human AQP1 (hAQP1), as an exemplar. Work here evaluated the fidelity of the yeast system in reproducing the full range of known hAQP1 channel properties, including regulation by intracellular messenger and protein kinase signaling pathways, sensitivity to pharmacological agonists and antagonists, modulation by divalent cations, and structure–function relationships assessed by results of site-directed mutagenesis. Outcomes here show the yeast expression method accurately recapitulated known hAQP1 channel properties, and thus has promise as a mass-throughput tool for screening novel AQPs as well as providing a platform for new discovery of pharmacological agonists and antagonists for AQPs.

In the five-pore AQP1 tetrameric channel, the four intrasubunit water pores selective for water and glycerol are physically and pharmacologically distinct from the central ion channel [14–16], which has enabled dissection of AQP1 water versus ion permeation roles. The intracellular loop between transmembrane domains 4 and 5 of hAQP1, known as loop D, is necessary for ion channel activation by cGMP; site-directed mutations of highly conserved arginine residues to alanine in loop D domain substantially impair cation channel activation [17,18]. The permeability of activated AQP1 ion channels to a range of monovalent cations, including lithium, allowed the use of an optical probe known as SHL (Sabrina Heng Lithium; a membrane permeable photoswitchable fluorescent Li^+^ indicator) for real time detection of AQP1 ion channel activity in mammalian cell membranes [19].

HgCl_2_ is a classic blocker of hAQP1 water pores which acts by covalently binding a cysteine residue (C189 in hAQP1) located in the outer vestibule of the water pore [20], without blocking the central pore ion conductance in wild-type AQP1 [14]. Forskolin, a stimulator of adenylate cyclase, was the first agent reported to activate the ion conductance of hAQP1 channels expressed in *Xenopus* oocytes, acting indirectly via a protein-kinase-A (PKA) dependent mechanism that was blocked by the non-selective protein kinase inhibitor H7 [21]. Direct binding of intracellular cGMP to the gating domain (cytoplasmic loop D) was subsequently identified as the primary mechanism of ion channel activation of AQP1 [18,22], independent of PKA or protein kinase G. Ion channel activity of hAQP1 was found to be enhanced by protein kinase C (PKC)-mediated phosphorylation of threonine residues in gating loop D and the C-terminal domain [23]. Regulation of the availability of hAQP1 channels to be gated by cGMP was linked to tyrosine kinase phosphorylation of the C-terminal domain [14].

Similar to channels and transporters, subsets of AQP classes are regulated by various factors including pH [11,24–27], phosphorylation [12,28], osmotic pressure [29,30], divalent cations [31,32] and other parameters. Discovery of new AQP ion channels and their activators and inhibitors could inform design of innovative therapies for diseases such as cancer, sickle cell anemia, edema, and others which have been linked to AQPs [33–36]. Elucidated with the yeast screening method, testing of genetic modifications and pharmacological tools could offer insights into consequences of inherited mutations, and improved interventions for diverse challenges ranging from engineering drought tolerance in crops [2] to controlling human glioblastoma invasiveness [37,38]. Unbiased surveys of diverse classes of AQPs tested in parallel against a matrix of regulatory signaling agents will be a valuable goal for future work.

## Materials and methods

### Gene subcloning and transformation into yeast

The open reading frame sequence of hAQP1 (NP_932766.1) in the mammalian expression vector pCDNA3.1 was obtained from GenScript. Full-length AQP cDNAs with and without stop codons were amplified from the pCDNA3.1 vector by PCR using Platinum™ PCR SuperMix High Fidelity (Thermo Fisher Scientific) and gene-specific primers, and subcloned into the Gateway entry vector pENTR/D-TOPO (Thermo Fisher Scientific). Recombination used the LR Clonase II Enzyme Mix (Thermo Fisher Scientific). Plasmids were transformed into chemically competent DH5-α *E. coli* and transformants selected with appropriate antibiotic selection markers. Plasmid DNA from transformants isolated using GenElute Plasmid DNA Miniprep Kit (Merck) was sequenced prior to transformation into yeast. Site-directed mutagenesis of AQP sequences was performed using the QuickChange II Site-Directed Mutagenesis Kit (Agilent). For localization in CY162 cells, hAQP1 was tagged with GFP (at the N-terminus) or DsRed (at the C-terminus) using destination vectors pAG426GAL-EGFP-ccdB and pAG423GAL-ccdB-DsRed respectively (Addgene, Susan Lindquist collection, MA U.S.A.).

For yeast mass-throughput screening assays, commercially synthesized (Genscript) coding sequences of genes for *hAQP1* and *AtKAT1* (*Arabidopsis thaliana* inward rectifier potassium channel) were subcloned into the high-copy number pYES-DEST52 vector (Invitrogen) downstream of the galactose-inducible *gal1* promoter. Yeast cells were grown to early logarithmic phase, and plasmids were transformed into yeast using the LiAc method [39]. For screening assays, wild-type and mutant AQPs were transfected into two strains of yeast, lacking K^+^ influx transport, or lacking endogenous native AQPs. The yeast strains used in the present study (Table 1) were the K^+^ uptake-deficient yeast mutant strain CY162 (Δ*trk1*, Δ*trk2*) [40], and a strain lacking native yeast AQPs, *aqy-null*, (Δ*aqy1* Δ*aqy2*) [30]. The strain CY162 (MATα ura3-52 his4-15 trk1Δ trk2Δ1::pCK64) was obtained from the National BioResource Project, Japan (https://yeast.nig.ac.jp/yeast/). The native AQP-deficient strain *aqy-null* (MATa leu2::hisG trp1::hisG his3::hisG ura3-52 aqy1::KanMX4 aqy2::HIS3), a derivative of *S. cerevisiae* 10560-6B [30], was kindly provided by Dr Grozman (Australian National University, Australia). Yeast cells were grown to early logarithmic phase in Yeast Extract-Peptone-Dextrose (YPD) media for aqy1aqy2 or YPD+100 mM KCl for CY162, and transformed by the lithium acetate method [39]. AtKAT1 served as the positive control in CY162 tests.

**Table 1:** Summary of yeast strains used in the present study

Strainṭ	Reference	Phenotype	Genotype
CY162	Ko and Gaber et al. (1991) [91]	K^+^ sensitive	MATα ura3-52 trk1Δ his3Δ200 his4-15, and trk2Δ:: pck64
aqy1aqy2	Leitão et al. (2014). [30]	Osmotic stress sensitive	Mat α; leu2::hisG; trp1::hisG, his3::hisG; ura352 aqy1D::KanMX aqy2D::KanMX

### Yeast cell culture and growth assays

Yeast strains were propagated aerobically at 29 °C in Yeast Nitrogen Base (YNB) medium (∼7.5 mM KCl, 6.7 g/l yeast nitrogen base without amino acids, 2% glucose, 2 g/l uracil-dropout amino acid mixture, supplemented with 100 mM KCl). Growth was monitored by optical density in liquid cultures. Growth assays for the CY162 strain were done in K^+^ -free YNB (6.7 g/l yeast nitrogen base without amino acids, 2% glucose, 2% raffinose, and 1.98 g/l uracil-dropout amino acid mixture; with 2% agar for solid media) supplemented with KCl as indicated. Growth assays with the *aqy-null* strain were done in normal YNB (∼7.5 mM KCl, 6.7 g/l yeast nitrogen base without amino acids, 2% glucose, 2% raffinose, and 1.98 g/l uracil-dropout amino acid mixture; with 2% agar for solid media). For osmotic stress experiments, growth media were supplemented with indicated concentration of NaCl, KCl or osmoequivalent concentration of sorbitol [41]. Cells were grown on YNB plates (no additional KCl) for *aqy1aqy2* deficient cells, and on YNB plates with 100 mM KCl for CY162 cells. Unbuffered medium pH ranged ∼4.2 ± 0.2. For media with defined acidic pH values (5.0, 5.7 or 6.4), pH was buffered using MES (2-(N-morpholino)ethanesulfonic acid) and Tris base to final values. For pH 7.3,Tris base without MES was used. A starting cell density (∼0.005–0.01 cells/200 µl) was found to give reproducible results for growth rate assays in 96-well plates.

### Subcellular localization of fusion protein hAQP1 channels in yeast

CY162 cells expressing fusion proteins (GFP-hAQP1; hAQP1-DsRed), or DsRed cytosolic marker alone (pYES-DEST52-DsRed) were grown to stationary phase (OD600 ∼ 2.0–3.0), washed, resuspended in inducing medium (∼1:10) and grown again to stationary phase (∼48 h). Small volumes (5 μl) of yeast cell cultures on polylysine coated slides (Thermofisher Scientific) were enclosed under coverslips with perimeters sealed by clear nail varnish. Fluorescence signals were visualised with a Leica TCS SP5 laser-scanning confocal microscope (Leica, Wetzlar, Germany) for eGFP (Excitation 488 nm; Emission 495–570 nm) and DsRed (Ex: 558 nm; Em: 584–645 nm). Hoechst 33258 (cat # 861405; Sigma-Aldrich, St. Louis, MO) staining (10 min at room temperature, 1:1000 dilution) showed cell nuclei (Ex: 405 nm; Em: 461 nm). Cell membranes were stained with MemBrite™ (green or red) as per manufacturer’s instructions (Ex: 488 nm; Em: 513 nm for green; or Ex: 561 and 603 nm for red). Fiji (ImageJ) software (U.S. National Institutes of Health) was used to quantify signal intensities as previously described [19].

### Optical fluorescence imaging of cation permeation in hAQP1-expressing yeast

The lithium sensitive photoswitchable probe (known as SHL) was prepared as described in our previous study [19]. For cell preparation, empty vector and AQP1-expressing CY162 cells were initially grown overnight in liquid YNB, containing 2% glucose supplemented with 100 mM KCl. Cells were washed 2–3 times with autoclaved MilliQ-filtered (MQ) water and aliquots were inoculated (OD600 ∼0.2–0.5) into induction culture medium (potassium free YNB, uracil, 2% galactose, 2% raffinose, 100 mM KCl). Cell cultures were incubated on a rotary shaker for 48 h (250 rpm, 29 °C) to allow time for protein expression. For confocal imaging, cells were spun down and washed 2–3 times with MQ water, resuspended in buffered saline containing LiCl (1.8 M sorbitol, 50 mM LiCl, 5 mM CaCl2, 10 mM Tris adjusted to pH 7.4 with HCl), and transferred into uncoated Ibidi 8-well slides (Ibidi, Munich, Germany). Slides were protected from light with aluminum foil prior to imaging with a Leica laser scanning confocal microscope (Wetzlar, Germany). Images were taken after 10 min exposure to white light (shifting SHL to the OFF-state) to measure the background signal, and after 10 min exposure to UV (632 nm) to restore SHL to the Li^+^-activatable conformation (ON-state) capable of monitoring cation permeation (Ex: 514 nm; Em: 550–650 nm). Fluorescence intensities were quantified with Image J software (National Institutes of Health, MD U.S.A.).

### Voltage clamp analyses of hAQP1 channels expressed in Xenopus oocytes

Partial ovariectomy surgery (done in the Laboratory Animal Services Facility, Helen Mayo Building, University of Adelaide) was used to harvest samples of unfertilized oocytes from adult female *Xenopus laevis* fully anesthetized by 8 min immersion in 0.2% Tricaine (MS-222; Merck, New South Wales Australia) w/v in charcoal-filtered tap water, pH 6.5, in accord with Care and Use of Laboratory Animals guidelines using protocols approved by the University of Adelaide Animal Ethics Committee (M-2018-016). Collagenase IA was used to defolliculate oocytes; stage V-VI oocytes were manually selected and injected with 50 nl of sterile water containing ∼1 ng of hAQP1 cRNA using a microinjection pipette (Drummond Scientific, Broomall, PA, U.S.A.). cRNA for hAQP1 (NCBI # NM_198098.3) was synthesized from NheI-linearized pGEMHE plasmid with the mMessage mMachine T7 kit (Ambion) using published methods [11,22]. Control non-AQP1-expressing oocytes were injected with 50 nl of sterile water or were not injected. Oocytes were incubated in standard Frog Ringers saline (mM: 100 NaCl, 2 KCl, 0.6 CaCl_2_, 5 MgCl_2_, 5 HEPES, with 100 U/ml penicillin, 100 µg/ml streptomycin, 0.5 mg/ml tetracycline and 5% (V/V) horse serum (Merck); pH 7.6) for 2-3 days to allow protein expression. Successful expression of hAQP1 in the oocyte plasma membrane was confirmed by osmotic swelling assays.

Two-electrode voltage clamp was used to record currents from control and hAQP1-expressing oocytes at room temperature in isotonic K^+^ saline (100 mM KCl, 1 mM NaCl, 1 mM MgCl_2_, 5 mM buffer). Buffers used were MES for final pH 5.1 and 6.1, or Tris Base for pH 7.4. Capillary glass electrodes (1-3 MΩ) were backfilled with 1 M KCl. Before use, oocytes were rinsed in Ca^2+^ free isotonic K^+^ saline. Recordings were done using a GeneClamp 500B amplifier (Molecular Devices, CA, U.S.A.), with Clampex 9.0 software (Molecular Devices, California, U.S.A.). AQP1 ion channels were activated with CPT-cGMP (8-(4-chlorophenylthio)guanosine 3′,5′-cyclic monophosphate; Merck), a membrane permeable form of cyclic GMP, added to bath saline at a final concentration of 20 μM. Current amplitudes before and after cGMP were monitored for 30 minutes using repeated steps to +40 mV (800 ms duration, 6 s interpulse interval) from a holding potential of −40 mV. CdCl_2_ (600 µM in bath saline) was used to inhibit the hAQP1 ion conductance. Conductance values were calculated from slopes of linear fits of current amplitude plotted as a function of voltage using GraphPad Prism 9 software, and compiled in histograms for statistical analyses.

### hAQP1 expression and ion channel function in *E. coli*

*E. coli* TK2463 defective in K^+^ uptake [42] was used to independently evaluate hAQP1 ion channel activity. cDNA subcloned into a modified pET-DEST42 expression plasmid (Thermo Fisher Scientific) by LR recombination was confirmed by sequencing; plasmid modification involved replacing the T7 promoter with the isopropyl β-D-1-thiogalactopyranoside (IPTG) inducible TAC promoter. Plasmids were transfected into chemically competent TK2463 cells (*F*^−^ thi lacZ amx82 rha Δ*[trkA] trkD1* Δ*[Kdp-FAB]5 endA*) by heat shock [43,44]. Luria broth (LB) was supplemented with 2 mM KCl (final concentration ∼4 mM KCl). Overnight *E. coli* cultures were washed 2-3 times in standard LB and resuspended. For screening in Corning 96-well microplates, E. *coli* (starting density 0.1 OD_600_) were inoculated into media (200 μl per well) supplemented with carbenicillin (100 μg/ml), with or without CPT-cGMP (20 µM), with or without 0.5 mM IPTG. Plate lids were replaced with MicroAmp Optical Adhesive Film (Thermo Fisher Scientific). OD_600_ values were measured every 30 min with a FLUOstar Omega Fluorescence microplate reader (BMG LABTECH) for 40 h at 37°C, with continuous double orbital shaking at 200 rpm to preclude sedimentation and cell clumping.

## Results

### Rescue of growth by cation channel expression in K^+^-transport-deficient CY162 yeast

**Figure 1:**
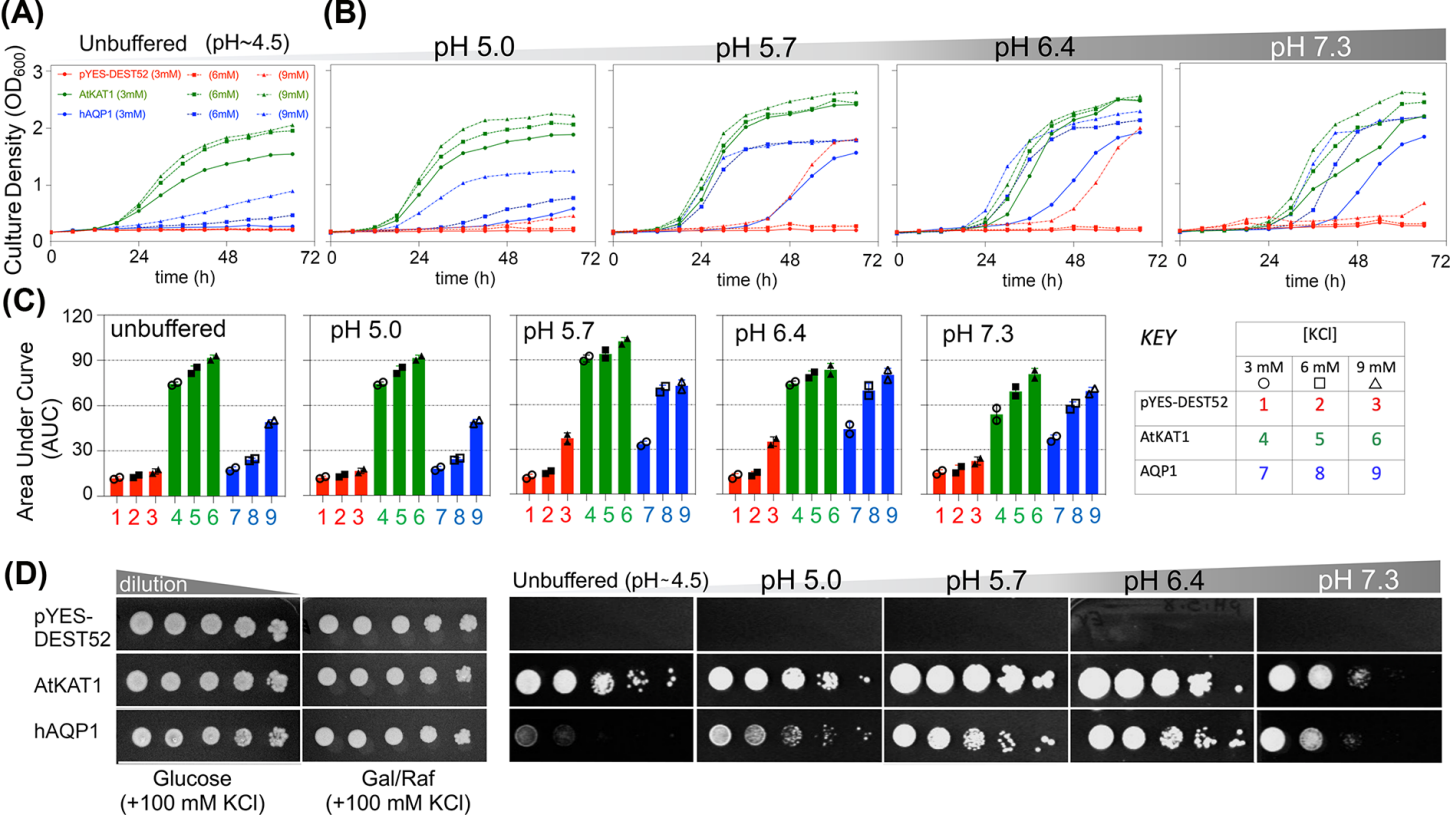
Complementation of growth in K^+^-uptake deficient CY162 yeast by transfection with hAQP1 K^+^ uptake enabled by heterologous hAQP1 expression yielded concentration-dependent rescue of growth in CY162 yeast cells. hAQP1-expressing yeast were grown in liquid culture (200 µl/well) in (**A**) unbuffered (∼pH 4.5) inducing medium or (**B**) in inducing media adjusted to pH 5.0, 5.7, 6.4, or 7.3. Growth was monitored in 96 well plates by optical density at 600 nm (OD_600_) as a function of time in media supplemented with KCl at 3 mM (circle), 6 mM (square), or 9 mM (triangle symbols). Mock transfected yeast (pYES-DEST52, red) was the negative control; CY162 yeast expressing the K^+^ channel AtKAT1 (green) was the positive control. (**C**) Growth responses quantified as calculated Area Under Curve (AUC) values were compiled in histograms showing mean ± SD for two independent experiments with three replicates each. (**D**) Phenotypic assays on solid media showed colony growth of hAQP1 and control lines, aliquoted from stock (OD_600_ = 1.0) using 10-fold serial dilutions on low-salt yeast nitrogen base medium supplemented with 6 mM KCl, incubated for 5 days. Yeast expressing hAQP1 or AtKAT1 showed strong growth in standard media (right panels); supplementation with high (100 mM) KCl allowed growth of negative control as well (left panels).

The expression of AtKAT1 and hAQP1 conferred dose-dependent rescue of K^+^ uptake-deficient CY162 yeast growth at extracellular K^+^ concentrations from 3 to 9 mM KCl (Figure 1). AtKAT1 expressing cells (positive control) showed the steepest growth rates and shortest latency to onset of exponential growth phases in both unbuffered (Figure 1A) and buffered media (Figure 1B). Similarly, hAQP1 rescued CY162 growth at levels intermediate in amplitude and latency as compared with AtKAT1, but significantly greater than empty vector which yielded a small slow response with 9 mM K^+^ or no rescue at lower K+ concentrations (Figure 1B). The dose-dependent effect of extracellular K^+^ in enabling growth was robust and consistent for AtKAT1- and hAQP1-expressing cells across all pH values tested.

Total growth measured as Area Under the Curve (AUC) from plots of optical density vs time appeared to suggest minor effects of pH (Figure 1C), with AtKAT1 showing slightly reduced growth at neutral pH, in contrast to hAQP1-expressing cells which showed slightly reduced growth at the most acidic pH (pH ≤ 5.0). This was corroborated by growth on solid media with agar (Figure 1D). All CY162 colonies grew well on permissive solid media containing 100 mM KCl (Figure 1D, left panel). With 6 mM KCl, CY162 yeast expressing *hAQP1* or *AtKAT1* but not empty vector grew (Figure 1D, right panel). Optimal growth on solid media for both channel types was observed at pH 5.7 and 6.4, not at high or low pH, suggesting little difference in pH effects on ion channel properties (Figure 1D). This finding is consistent with electrophysiology studies of AQP1 ion channels expressed in *Xenopus* oocytes which showed little pH sensitivity (Supplementary Figure S1). An innate pH effect on yeast physiology also can be inferred from the observed empty vector growth responses in 9 mM KCl which were small but detectable at pH 5.7 and 6.4, not at pH 5.0 or 7.3 (Figure 1B).

### hAQP1 ion currents expressed in *Xenopus* oocytes show little pH sensitivity

Effects of pH on hAQP1 ion channel properties in the oocyte expression system were evaluated by two-electrode voltage clamp (Supplementary Figure S1), following established protocols [22]. Steady state current amplitudes measured for a series of voltage steps were plotted to allow calculation of whole cell conductance from slope values of linear regression fits of the current-voltage relationships (Supplementary Figure S1A). The hAQP1 ion current responses (Supplementary Figure S1B, left panel) were activated by stimulation with membrane permeable CPT-cGMP in each condition. Results showed no appreciable effects of pH from 5.1 to 7.4; AQP1-expressing oocytes displayed cGMP-dependent activation. Ion current responses to cGMP were not seen in non-AQP1-expressing control oocytes over the tested pH range (Supplementary Figure S1B, right panel). AQP1 channels showed expected properties of block by extracellular Cd^2+^, no appreciable voltage sensitivity (shown by the near linear current–voltage relationships), and no detectable pH dependence (mean conductance amplitudes for each treatment were comparable across pH conditions). Compiled histogram data for conductance values measured for hAQP1-expressing and control oocytes (Supp Fig. S1C) confirmed statistically significant activation by cGMP and block by Cd^2+^, and showed the absence of pH-induced effects on cGMP-activated conductance values for hAQP1. Results showed hAQP1 retained ion permeability over a range of pH values, in agreement with yeast screening results (Figure 1). Possible pH sensitivity of the magnitude of Cd^2+^ block of hAQP1 remains to be investigated. As summarized in the Introduction, other AQP classes do show pH-sensitive modulation of function, which will be of interest for comparative work on other candidate AQP ion channels in future yeast screening assays.

### AQP ion conduction not water fluxes are required for K^+^-sensitive yeast rescue

**Figure 2:**
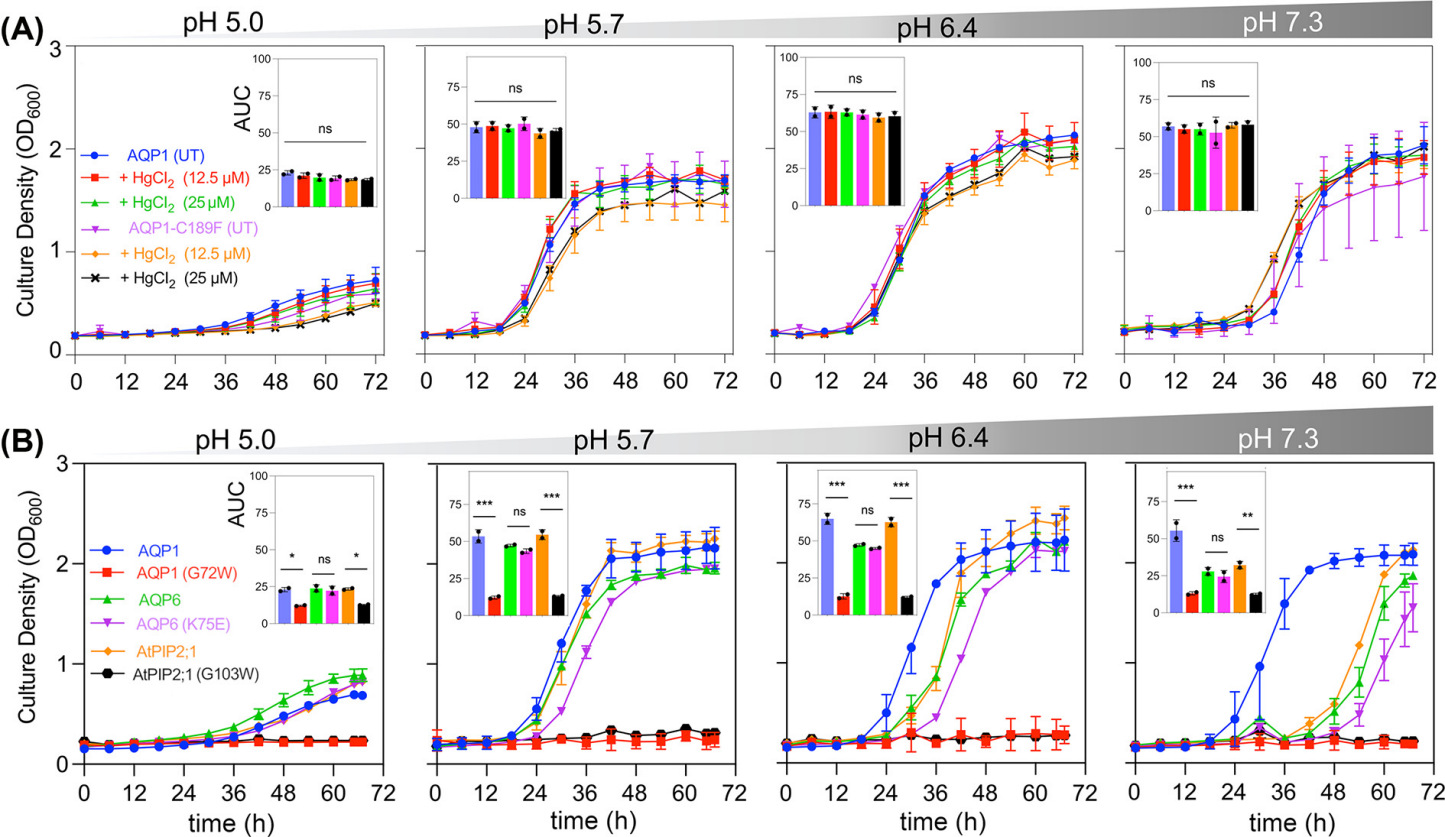
Rescue of yeast growth by AQP channel activity assessed by mercury sensitivity and effects of site directed mutations (**A**) hAQP1-mediated water permeability is not required for compensation of the CY162 yeast growth defect, as indicated by lack of sensitivity to the water pore blocker mercuric chloride (HgCl_2_) at 12.5 and 25 µM. Yeast expressing the Hg^2+^-insensitive hAQP1 mutant C189F showed growth not different from that in wild-type, with or without HgCl_2_ at any pH. (**B**) Three different classes of characterized AQP ion channels (mammalian AQP1 and AQP6, and plant AtPIP2;1) rescued CY162 growth at pH 5.7 and above in 6 mM KCl. Two mutations resulting in dysfunctional channels (hAQP1 G72W; AtPIP2;1 G103W) failed to rescue growth; whereas the mutant hAQP6 K75E which retains ion channel functionality supported growth not different from hAQP6 wild-type. Results compiled from two independent experiments (two replicates each) are quantified as AUC histograms (insets; mean ± SD). Statistical significance (ANOVA with post-hoc Bonferroni tests) is indicated as *P<0.05, **P<0.01, ***P<0.001; or ns (not significant).

Block of the AQP1 water pore with HgCl_2_ showed no effect on the rescue of yeast growth (Figure 2). CY162 cells expressing wild-type hAQP1 were not affected by HgCl_2_ at 12.5 or 25 µM (Figure 2A), consistent with the idea that water permeation is not necessary to compensate for the yeast growth defect. Mutation of hAQP1 C189 to phenylalanine (F) shown to eliminate water channel activity [45] similarly did not impair hAQP1-enabled yeast growth (Figure 2A).

Two other types of wild-type AQPs (AQP6 and PIP2;1) known to function as dual water-and-ion channels also were successful for rescuing CY162 growth in 6 mM K^+^ (Figure 2B). AQP6 channels have been shown to conduct anions after activation by Hg^2+^, mutation of lysine 75 to glutamate (K75E) in rat AQP6 shifted ion preference from anionic to cationic [25]. In tests with hAQP6, both wild-type and K75E mutant channels rescued yeast growth (Figure 2B), suggesting cations might also permeate AQP6 even if at a low level. Precedent for mixed ionic selectivity in an AQP channel was established for AQP0, which conducts both anions and cations but shows an anionic preference [46].

Expression of the *Arabidopsis* dual water-and-ion channel AtPIP2;1 rescued yeast growth when using wild-type, but mutation of AtPIP2;1 glycine 103 to tryptophan (G103W; which impaired both water and ion permeability [11]) did not rescue yeast growth (Figure 2B). The equivalent mutation in hAQP1 *(*G72W) also prevented the rescue of yeast growth (Figure 2B). These results showed that growth rescue of CY162 by hAQP1 is independent of water transport and mimicked by AQP channels that show capacity for cation conduction. It is important to note that channels in yeast were tested without applied stimuli; hAQP1 was tested without added cGMP, and AQP6 was not activated by Hg^2+^, suggesting baseline levels of AQP channel activity in the yeast system are sufficient to rescue K^+^ dependent growth over the three-day assay duration.

Properties of aquaporin channel expression, correct functional assembly, and localization in yeast plasma membranes were demonstrated by showing a gain-of-function sensitivity to osmotic pressure stress in AQP-transfected cells, which was due to the aquaporin-mediated increase in membrane water permeability as shown in Supplementary Figure S2.

### Effects of pharmacological modulators of hAQP1 on yeast cell rescue

**Figure 3:**
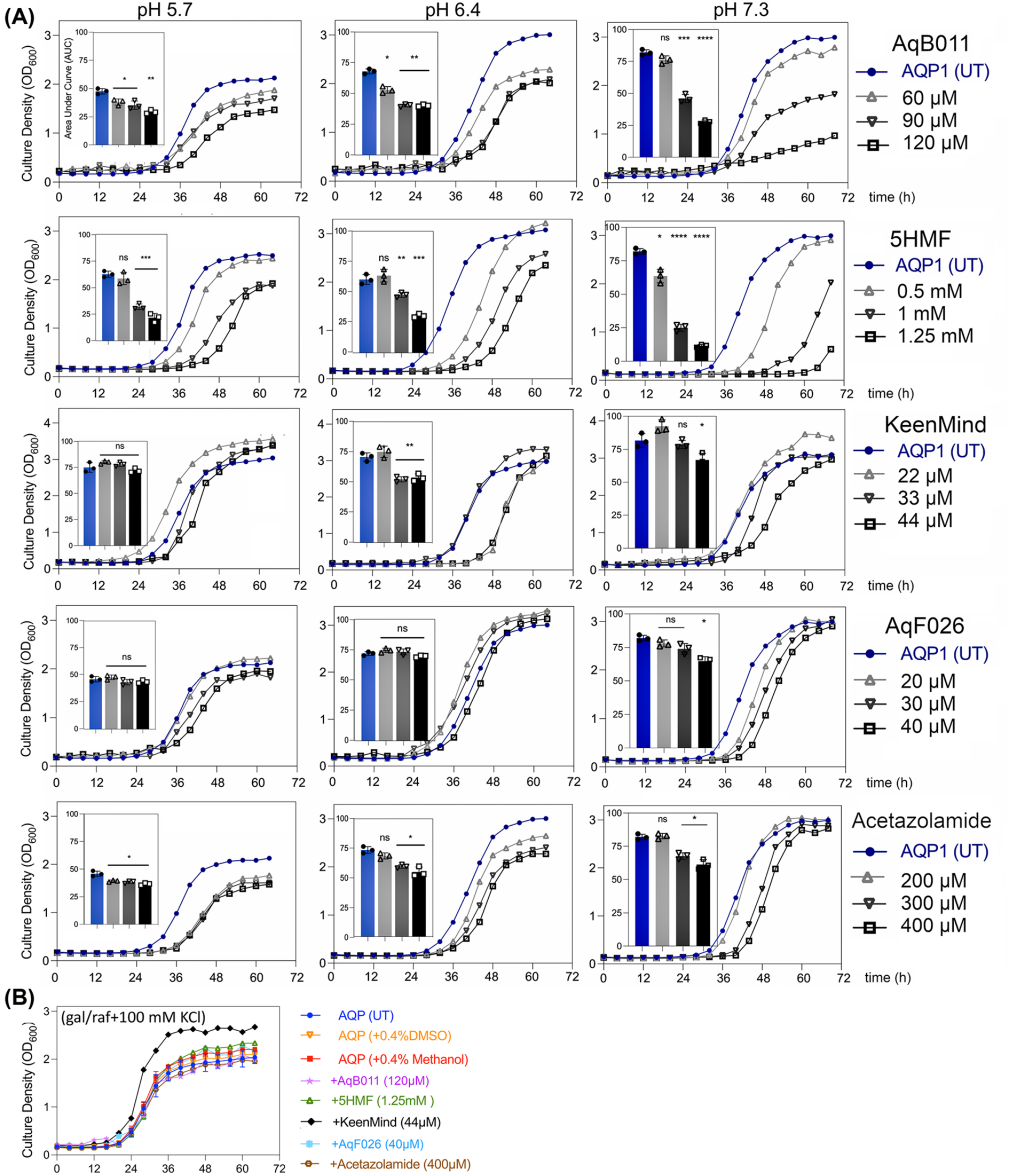
Assessment of pharmacological modulators of hAQP1 channel activity on yeast growth (**A**) Agents were tested for CY162 cell growth in media with 6 mM KCl at pH 5.7, 6.4, or 7.3, monitored for 68 h. AUC data histograms (insets) show results (mean ± SD) of two independent experiments each done in triplicate. Statistical significance compared with untreated AQP1 (unpaired *t-*test) is shown as ****P<0.0005, ***P<0.005, **P<0.05, *P<0.5, and ns, not significant. Vehicle controls matched to drug treatments as ‘untreated’ (UT) groups were: DMSO for AqB011, AqF026 and 5HMF; methanol for KeenMind; and water for acetazolamide. Data are compiled from two independent experiments with three replicates each; error bars show mean ± SD. (**B**) In permissive medium with 100 mM KCl, yeast expressing hAQP1 and drug treatments at the highest doses grew well on standard medium, showing absence of general toxicity.

Pharmacological modulators of AQP1 water and ion channel activities defined previously in mammalian and oocyte systems were tested here for the first time in yeast for effects on growth rescue in wild-type AQP1-expressing CY162 cells (Figure 3). CY162 yeast expressing wild-type hAQP1 were grown in inducing medium with and without pharmacological agents to test for dose-dependent effects. Agents included selective inhibitors of hAQP1 ion permeability (AqB011, 5HMF) [33,47], a combined inhibitor that decreases both water and ion permeability in hAQP1 (KeenMind), the selective hAQP1 water channel potentiator (AqF026) [48], and a proposed AQP water channel blocker acetazolamide. The botanic extract KeenMind contains the AQP1 water channel blocker bacopaside II as a major component and the AQP1 ion channel blocker bacopaside I as a minor component [49,50]. Acetazolamide is clinically important as an inhibitor of carbonic anhydrase at submicromolar concentrations [51], and also reported to inhibit water flux in some AQPs including AQP1 [36]. Strong dose-dependent block of growth was observed for hAQP1-expressing cells treated with the AQP1 ion channel antagonists AqB011 or 5HMF (Figure 3A, rows 1 and 2) at all pH values. KeenMind caused modest block of yeast growth at the highest dose (Figure 3A, row 3). AqF026 caused a limited effect, with a slight block only at the highest dose in the pH 7.3 condition (Figure 3A, row 4). Acetazolamide showed modest dose-dependent inhibition at pH 6.4 and 7.3 (Figure 3A, row 5), which could reflect enzyme inhibition, AQP inhibition, or both. At the highest doses, growth was not impaired in K^+^-rich standard medium (Figure 3B), indicating pharmacological agents did not indirectly induce cell death. The effectiveness of hAQP1 in rescuing yeast cell growth was not appreciably altered by pH, as seen in the comparable latencies to onset of exponential growth (∼26 h) across the untreated hAQP1-expressing groups (Figure 3A).

Although AQP1-rescued growth was not substantially influenced by pH in the range tested, it was interesting that pH did impact pharmacological effectiveness for two of the compounds, AqB011 and 5HMF, which were more potent at neutral than acidic pH values. With p*K*_a_ values near 13, both compounds are classified as very weak acids, indicating effects of proton titration on net charge (and thus membrane permeability) should be negligible. However, pH sensitivity of AqB011 and 5HMF inhibitory effects could reflect changes in AQP1 residues which alter activation or binding or could arise indirectly from pH-sensitive effects on the yeast itself, including for example altered lipid permeability, altered activity of drug transporter systems, or other possibilities that remain to be explored.

### Effects of intracellular signaling pathways that regulate hAQP1 ion channel activity

**Figure 4:**
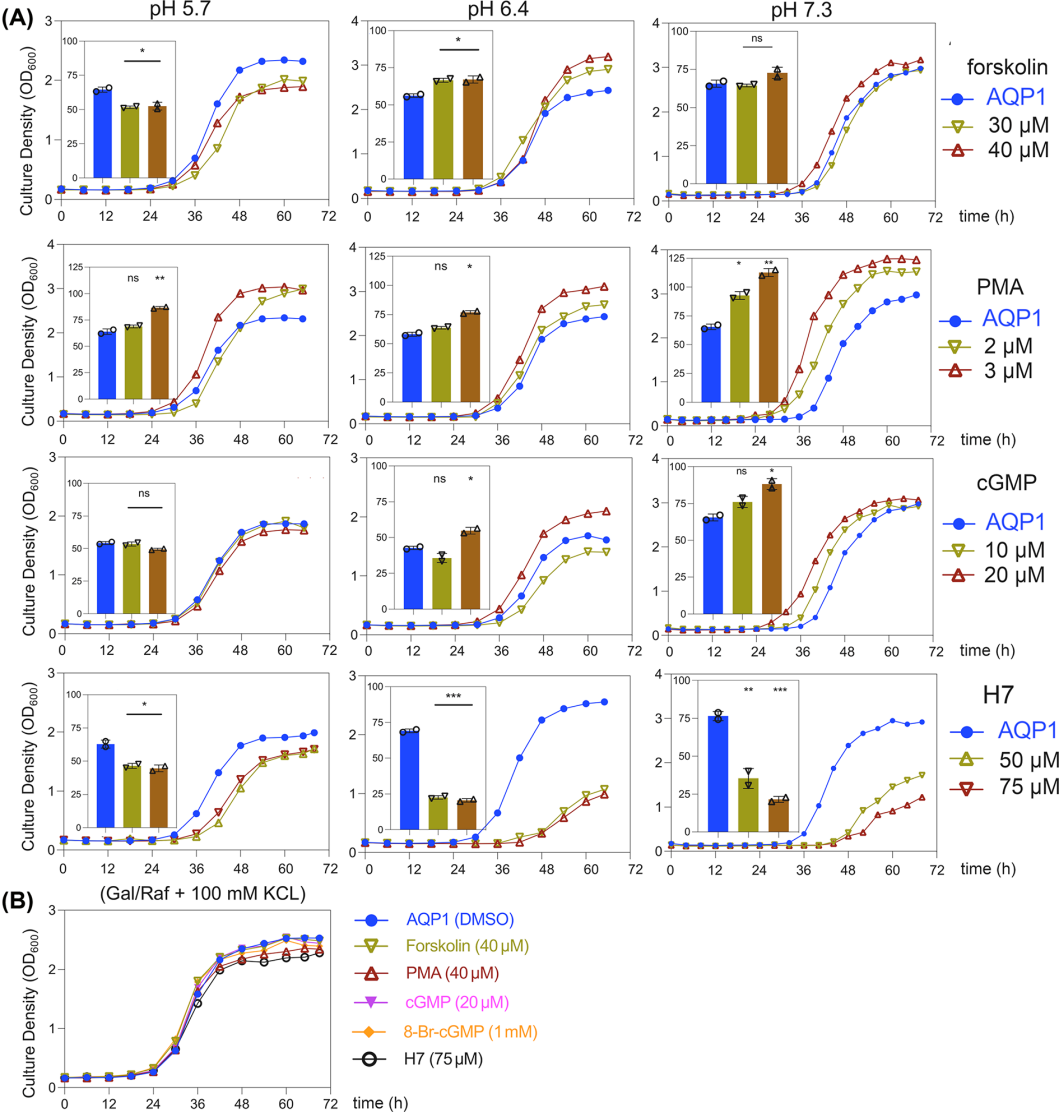
Regulation of hAQP1-induced growth rescue by modulators of intracellular signaling pathways (**A**) Growth responses for CY162 cells expressing hAQP1, grown in YNB inducing medium with 6 mM KCl at pH 5.7, 6.4 or 7.3. Vehicle (0.4% v/v DMSO) or water (UT) served as the matched control conditions for treatments with direct ligand (cGMP) and protein kinase modulating agents (forskolin, PMA, cGMP, and H7) as indicated. AUC summary data are shown in inset histograms. Statistical significance (unpaired T test) was determined by comparison of each treatment with matched vehicle or water controls, and shown as *P<0.05, **P<0.01, ***P<0.001; or ns (not significant). (**B**) Equivalent growth rates in permissive media (100 mM KCl) confirmed no toxic effects on growth for any of the treatments at maximal doses. All growth curves were compiled from two independent experiments, with three technical replicates each.

Intracellular signaling pathways reported previously to influence hAQP1 channel activation in other systems were tested for effects on yeast growth rescue in the CY162 strain (Figure 4).

The indirect AQP1 ion channel activator forskolin working via PKA showed a growth-stimulating effect on CY162 only at pH 6.4 (Figure 4A, row 1). The PKC activator, phorbol-12-myristate-13-acetate (PMA) showed dose-dependent potentiation of growth in all conditions, strongest at neutral pH 7.3 (Figure 4A, row 2). The membrane permeable analog of cGMP (CPT-cGMP) similarly caused dose-dependent facilitation of yeast growth at pH 7.3 (Figure 4A, row 3). Conversely as expected, the protein kinase inhibitor H7 inhibited growth particularly at pH 6.4 and 7.3 (Figure 4A, row 4). In summary, results in Figure 4A showed modulators predicted to increase hAQP1 ion channel activity facilitated growth rescue at mid-to-neutral pH; conversely, H7 which decreases hAQP1 ion channel activity suppressed yeast growth. At the highest doses used, none of the pharmacological agents altered growth in K^+^-rich standard medium (Figure 4B), ruling out indirect effects of cytotoxicity.

### Optical assessment of hAQP1 cation permeability in yeast plasma membrane

**Figure 5:**
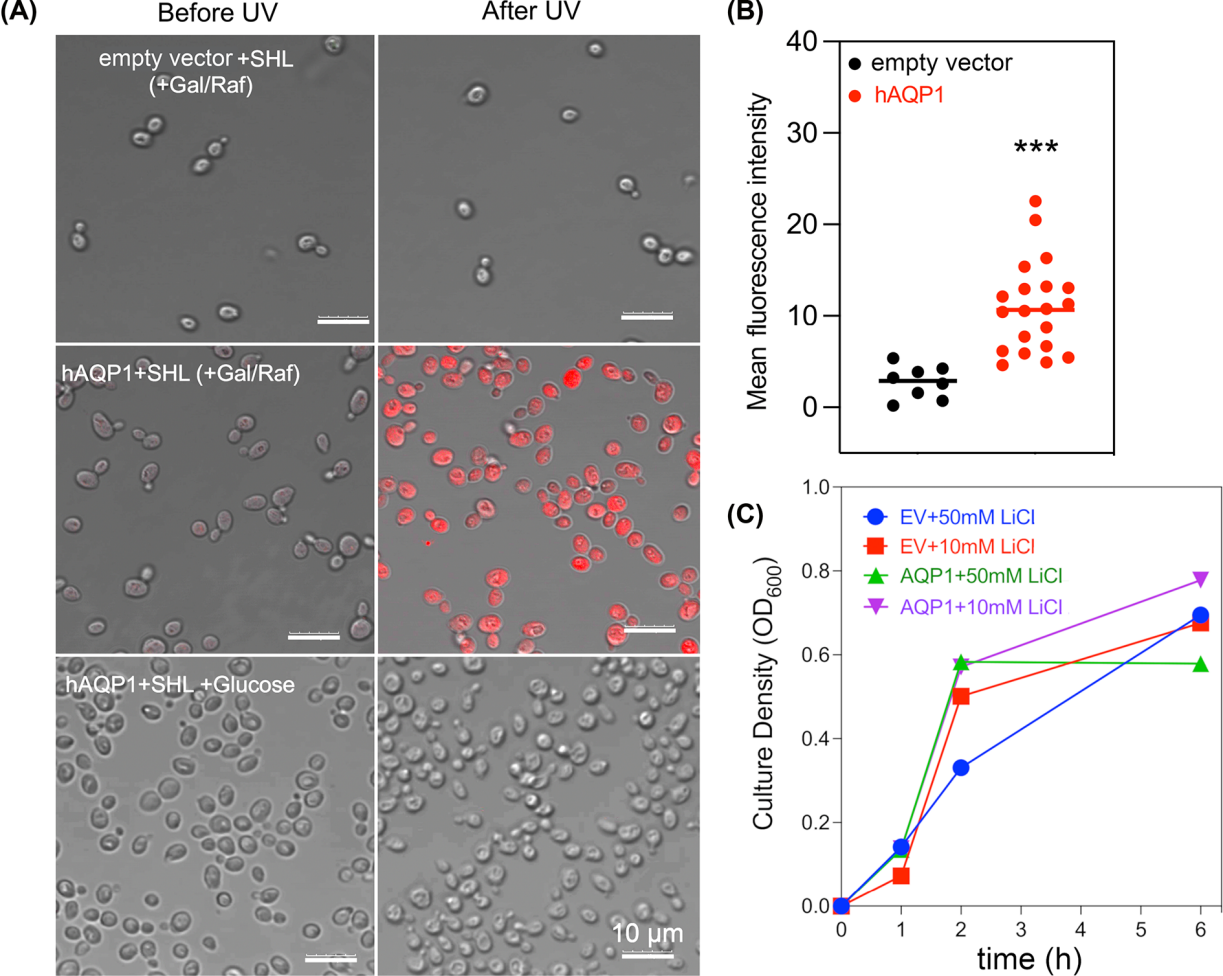
Optical confirmation of monovalent cation permeation in hAQP1-expressing yeast using a photoswitchable lithium sensor SHL (**A**) CY162 cells expressing pYES-DEST52 empty vector (EV) (upper row) or human (h)AQP1 (middle and lower rows) imaged by confocal microscopy with the Li^+^ sensor in the OFF (before UV) and ON (after UV) configurations, showing cation entry into cells induced to express AQP1 (middle row), not in non-AQP1 expressing controls (upper and lower rows). Li^+^ -bound SHL was detected using Ex 514 nm and Em 550-650 nm (see Methods for details). Controls were empty vector (upper low), and non-induced AQP1-transfected cells supplemented with 100 mM KCl (lower row). Scale bars are 10 µM. (**B**) Scatter plot of cellular fluorescence intensities for 8 empty vector control and 20 AQP1-expressing yeast cells which were induced in gal/raf with 6 mM KCl (as shown in A, middle row). Statistical significance determined by unpaired Student’s *t-*test shows ***P<0.0001. (**C**) Lack of effect of lithium exposure on yeast cell growth responses in hAQP1-expressing or empty vector CY162 cells induced in gal/raf at pH 7.3.

The function of hAQP1 as a monovalent cation channel in yeast was confirmed using a lithium-sensitive photoswitchable probe known as ‘SHL’ (Sabrina Heng Lithium) (Figure 5). This probe was designed as an optical tool for real-time detection of active hAQP1 ion channels in living cells, which are permeable to monovalent cations including Li^+^ [19,36]. Li^+^ entry into CY162 cells with empty vector (Figure 5A, row 1), and with induced expression of hAQP1 (Figure 5A, row 2), was compared with CY162 cells in which hAQP1 expression was not induced (Figure 5A, row 3). Images taken after white light exposure (SHL OFF-state; before UV) showed background levels, whereas after UV (SHL ON-state) Li^+^ signal was evident in AQP1-expressing cells, not seen in either set of control cells. Quantified fluorescence signal intensities were greater in hAQP1-expressing CY162 cells than non-induced control cells (Figure 5B). Results in Figure 5 confirmed ion channel activity in hAQP1-expressing cells and indicated native yeast membranes are relatively impermeable to Li^+^ in the absence of an introduced cation channel. Possible cytotoxicity due to acute Li^+^ exposure for CY162 cells reported to be sensitive to alkali metals [52] was ruled out by measuring growth with and without Li^+^ at the same dose and exposure duration used for SHL experiments, showing cells remained healthy (Figure 5C).

### Effects of divalent cations in blocking growth complementation by hAQP1

**Figure 6:**
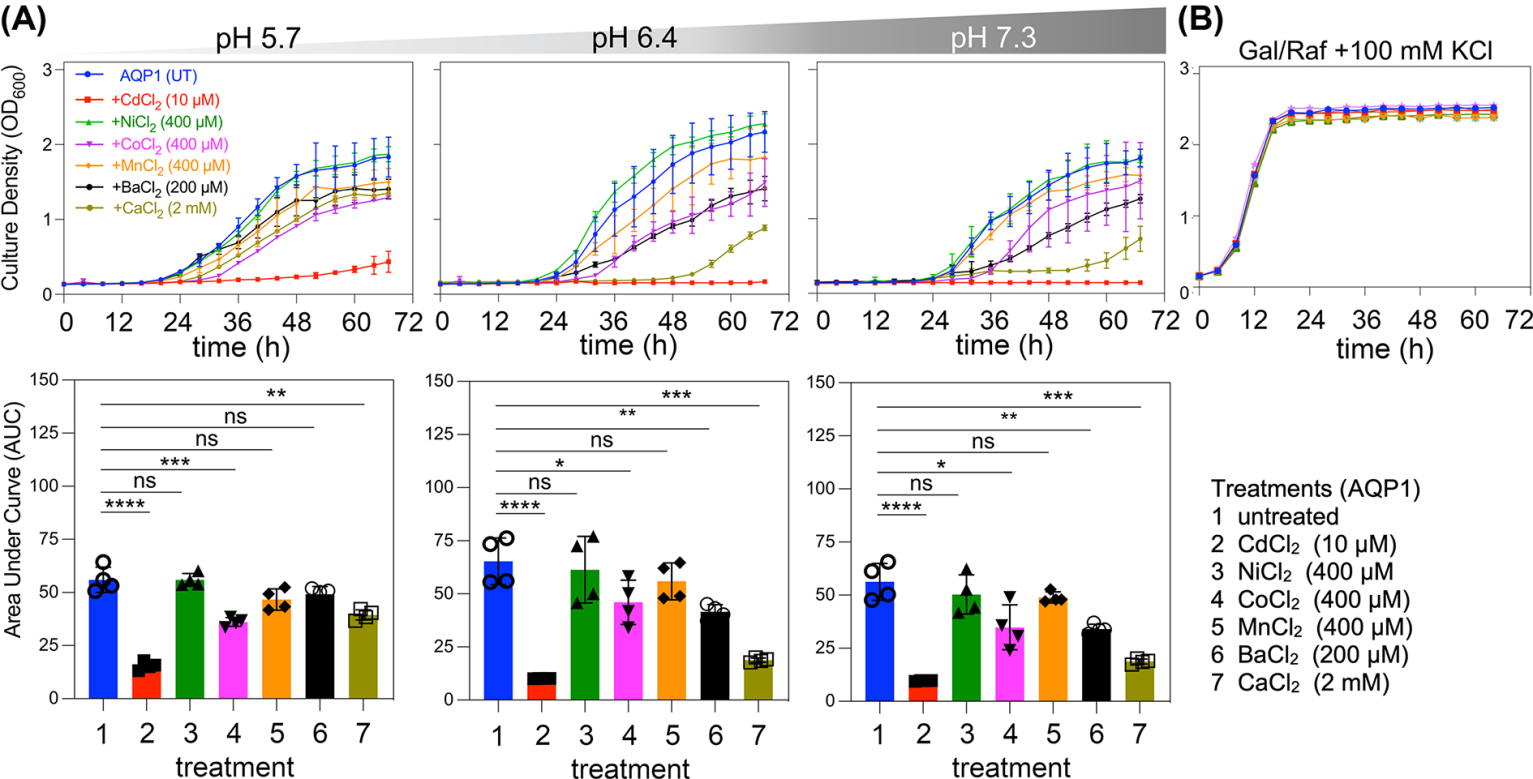
Effects of extracellular divalent cations on growth of hAQP1-expressing yeast (**A**) Effect of divalent cations on the growth of CY162 cells expressing hAQP1 at different pH values all with 6 mM KCl. Curves are shown for untreated hAQP1 (blue); Cd^2+^ (10 µM; red), Ni^2+^ (400 µM; green), Co^2+^ (400 µM; pink), Mn^2+^ (400 µM; orange), Ba^2+^ (200 µM; black) and Ca^2+^ (2 mM; sage). Growth rates compiled as AUC are shown below the curve plots as corresponding histograms, for data from least two independent experiments with two replicates each; error bars show mean ± SD. Statistical significance determined by ANOVA with post-hoc Bonferroni tests *P<0.05, **P<0.01, ***P<0.001; or ns (not significant). (**B)** Yeast expressing hAQP1 in permissive media with 100 mM KCl showed equivalent growth across treatments with or without divalent cations, indicating block was not due to general toxicity.

The ability of extracellularly applied divalent cations as Cl^−^ salts to differentially block the growth-enhancing effects of hAQP1 in CY162 yeast cells was tested at concentrations shown previously to inhibit AQP ion conductance, and extended to include three additional divalent species for comparison (Figure 6). Growth curves (Figure 6A, upper row) and compiled histograms for area under curve values (Figure 6A, lower row) showed the most effective blocker for hAQP1-expressing yeast growth was Cd^2+^ (10 µM), followed by Ba^2+^ (200 µM) and Ca^2+^ (2 mM), in accord with published data [31]. Not previously tested in AQP ion channels, Co^2+^ (400 µM) inhibited growth at all pH values, whereas Mn^2+^ or Ni^2+^ at 400 µM were ineffective. Inhibitory effects of divalents were not dependent on pH, apart from Ca^2+^ which appeared to show less block (i.e., higher growth and shorter latency) at pH 5.7 than at pH 6.4 or 7.3, which if verified might suggest a different site of action. Growth-inhibitory effects of divalent cations were not due to cytotoxicity; cell viability was not affected by the same concentrations of divalents applied in permissive 100 mM KCl medium (Figure 6B).

### Plasma membrane trafficking of hAQP1 is necessary to rescue yeast growth

**Figure 7:**
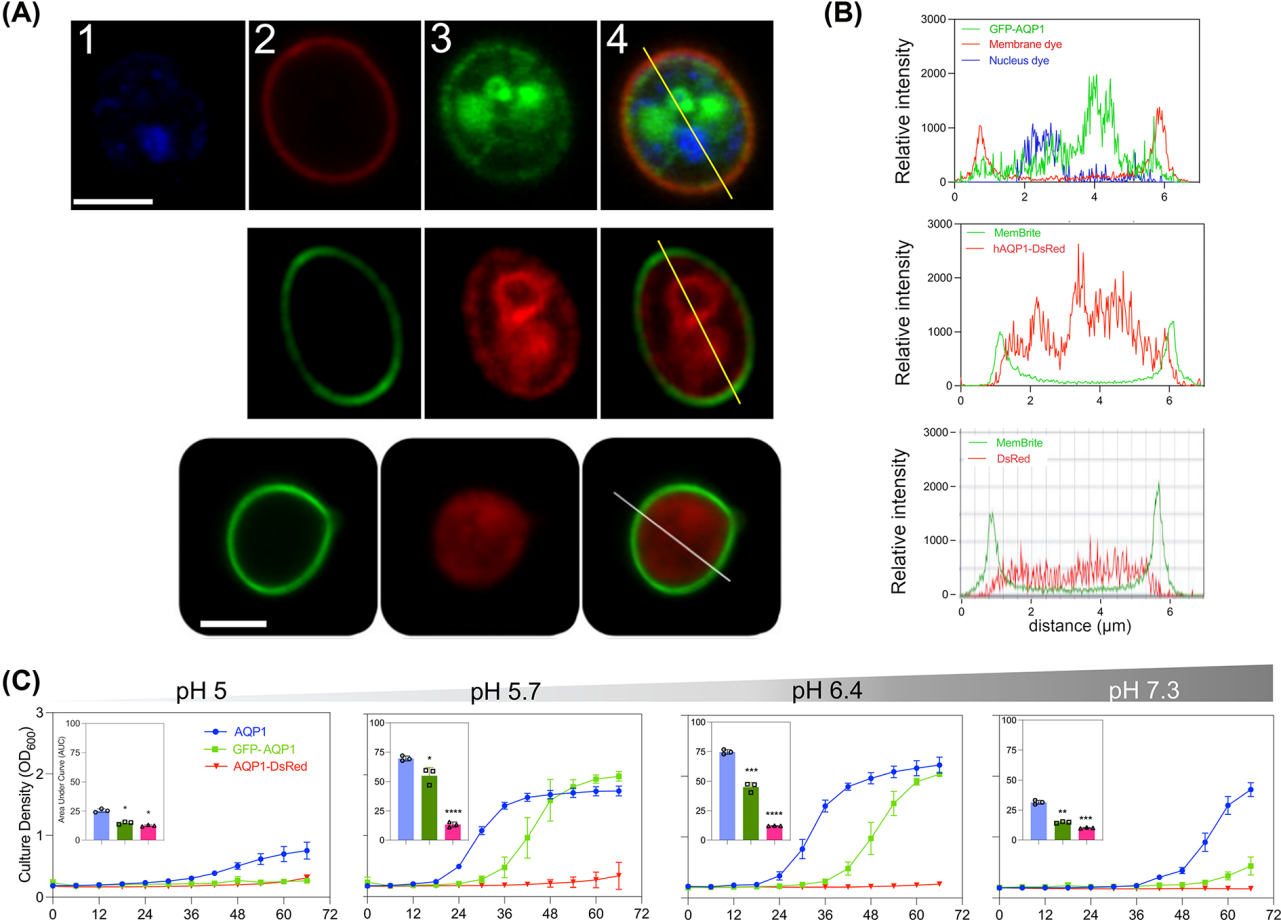
Fusion-protein tagging of hAQP1 influences subcellular localization and cation-dependent growth rescue in yeast (**A**) Live cell confocal microscopy images of CY162 cells expressing GFP tagged (upper row) or DsRED tagged (middle row) hAQP fusion proteins, or cells expressing fluorescent tag alone (lower row). Hoechst nuclear stain (1); plasma membrane dye (2; red upper row; green middle row); tagged GFP-hAQP1 (3; upper row) and tagged hAQP1-DsRed (middle row); cytoplasmic tag alone (lower row); and merged images (4). Scale bars 5 µm. Cells were induced in gal/raf with 6 mM KCl for ∼70 hours before imaging. (**B**) Lines in panel A show cross sections taken through cell centers to measure signal intensities for tagged hAQP1 or DsRed alone, with membrane dye and nuclear dye levels as indicated, plotted as a function of X-Y distance to determine overlap between fusion protein and plasma membrane localization. (**C**) Growth responses of CY162 cells transformed with hAQP1 (blue), GFP-hAQP1 (green), or hAQP1-DsRed (red) in media with 6 mM KCl at ∼70 hours, with pH (5.0-7.3). Compiled AUC values from three independent experiments (2 replicates each) were assessed by unpaired Student’s *t*-test showing *P<0.05, **P<0.01, ***P<0.001, ****P<0.0001; or ns (not significant).

hAQP1 channels were modified to create N-terminal GFP and C-terminal DsRed fusion protein constructs used to test the correspondence between growth rescue and plasma membrane localization (Figure 7). Red and green fusion protein-tagged hAQP1 channels in CY162 yeast were visualized by laser-scanning confocal fluorescence microscopy (Figure 7A), in combination with complementary color markers for plasma membranes, and blue Hoechst stain for cell nuclei. Cross-sectional plots of signal intensities (Figure 7B) showed that a small proportion of the tagged fusion-protein channels overlapped with the plasma membrane marker, indicating membrane colocalization for N-terminal tagged GFP-hAQP1 (upper row) and C-terminal tagged hAQP1-DsRed (middle row), not seen in yeast transfected with DsRed vector alone in which a uniform cytoplasmic distribution did not overlap with membrane (lower row). Consistent with low levels of fusion proteins in plasma membranes, z-stack analyses showed the signal distributions were mainly intracellular, appearing to be associated with endoplasmic reticulum and perinuclear regions (Supplementary Movies S1 and 2).

In growth assays with CY162, N-terminal tagged GFP-hAQP1 construct enabled moderate rescue of growth at pH 5.7 and 6.4 (Figure 7C), with responses similar in final amplitude to wild-type though longer in latency. In contrast, C-terminal DsRed fusion protein was ineffective in all pH conditions (Figure 7C), which might be due to poor membrane localization but also could reflect interference of the C-terminal modification with ion channel function, as supported by prior studies of mutations in C-terminal domain residues of hAQP1 expressed in oocytes that impaired ion channel activation but not water channel activity [53].

Plasma membrane localization of hAQP1 fusion proteins was corroborated by an osmotic stress assay with the yeast strain *aqy1aqy2*, which lacks native AQPs [41]. The presence of aquaporin channels reduces osmotic stress tolerance in yeast [41,54]. Both N- and C-terminal hAQP1 fusion proteins conferred enough water transport to replicate effects of wild-type hAQP1 in terms of causing failure of the transformants to grow under hypertonic osmotic stress conditions imposed by addition of 1.75 M KCl or 0.5 M NaCl (Supplementary Figure S2). These results confirmed that both AQP1 fusion proteins retained water channel activity, showing they were trafficked to yeast plasma membrane albeit at low levels, but still sufficient to impact phenotypic properties. These data suggest that poor growth rescue by C-terminal hAQP1-DsRed was due to compromised ion channel activity rather than complete absence of membrane expression, providing additional evidence that ion channel function of hAQP1 is required for effective rescue.

### Structure–function analyses of mutant hAQP1 channels in yeast growth rescue

Site-directed mutations in gating loop D and transmembrane domains that alter hAQP1 function, or are equivalent to mutations known to affect water or ion channel properties in other AQP classes (summarized in Table 2) include the double mutant R159A+R160A, and single mutants D158P, R160P, G166P, E17N, G57N and G72W. These hAQP1 mutants were transfected into CY162 yeast to test effects on growth rescue as compared with wild-type hAQP1 (Supplementary Figure S3). Strong reductions in growth were imposed by R159A+R160A and D158P at pH 5.7 and 6.4, with near complete suppression of rescue evident at pH 7.3. The mutations R160P and G166P showed mild reduction of growth at pH 5, and no effect at pH 6.4 or 7.3. For these four mutant constructs, the magnitude of growth suppression in yeast was correlated with the loss of ion conductance in prior voltage clamp studies of oocytes (Table 2). R159A+R160A, D158P, R160P and G166P constructs were shown in prior work to be membrane-localized as confirmed by retention of osmotic water channel activity in oocytes or by immunocytochemistry (Table 2), supporting the interpretation that mutations which compromised AQP ion channel function were associated with the loss of yeast growth rescue. Three mutant constructs eliminated growth rescue in all pH conditions, E17N, G57N and G72W. In the two Gly mutation constructs, the cause can be attributed to be poor membrane expression, technically making these sham controls. Mutated to mimick AQP6 wild-type (N60), the construct AQP1 G57N did not traffic to plasma membrane [55]. The plant AtPIP2;1 equivalent (G103W) of AQP1 G72W showed a minor contribution to cellular water channel activity only when coexpressed with correctly targeted subtypes [11]. However, the insect BIB equivalent (E71N) of AQP1 E17N lacked ion channel activity but was trafficked to plasma membrane in oocytes [56][92].

**Table 2:** Site-directed mutations known to alter HsAQP1 ion channel properties, or predicted to impair function based on equivalent mutations in other AQPs

hAQP1 mutation	Structural location	Effect(s) on channel function (as compared with wild-type)	References
R159A+R160A	loop D gate	Slowed response to cGMP and impaired ion conductance in HsAQP1 R159A+R160A (reduced by ∼60–80%) Normal water channel activity	Kourghi et al., 2018. [17] Yu et al., 2006. [18]
D158P	loop D gate	Significant block of hAQP1 ion channel activation (reduced to ∼10%) Normal water channel activity	
R160P	loop D gate	Decreased ion conductance of hAQP1 (to ∼30%) Normal water channel activity	
G166P	loop D gate	Enhanced amplitude conductance response after cGMP stimulation (∼4-fold increase) Normal water channel activity	
E17N	TM1	Knockout of AQP ion channel function in *Drosophila* BigBrain (Dm BIB E71N). ICC confirmation of plasma membrane expression Loss of water and reduction of ion channel activity by an equivalent mutation in AQP1 (Hs AQP1 E17N)	Yanochko and Yool, 2002. [56] Yool. 2007. [92]
G57N	TM2	Decreased water flux in RnAQP1 G57N (to ∼10% of wild-type), attributed to failed membrane targeting of channels	Liu et al., 2005. [55]
G72W	water pore	Knockout of AQP ion channel function in *Arabidopsis* AtPIP2;1 G103W. Partial water channel activity retained in a coexpression assay	Byrt et al., 2017. [11]

Dm = *Drosophila melanogaster*; Hs = *Homo sapiens*; ICC = immunocytochemistry; Rn = *Rattus norvegicus*; TM = transmembrane domain.

Plasma membrane expression levels in yeast for hAQP1 mutants R159A+R160A, E17N and G72W mutants were evaluated using osmotic stress assays with the *aqy-null* strain (Supplementary Figure S2A). Osmotic stress imposed with 1.75 M KCl or 0.5 M NaCl impaired growth for the R159A+R160A and E17N constructs, indicating these channels conferred osmotic water permeability and thus were assembled and trafficked to plasma membrane. In contrast, effects of G72W were not different from empty vector (Supplementary Figure S2A), supporting the interpretation that lack of rescue in this case was consistent with general disruption or impaired membrane trafficking, similar to G57N. None of the constructs introduced into yeast cells prevented growth in permissive high K^+^ medium (Supplementary Figure S2B), indicating that yeast viability was not compromised indirectly by transfection or exogenous protein expression.

### hAQP1-enabled rescue of growth in a potassium transport-deficient *E. coli* strain

The TK2463 *E. coli* strain lacks major K^+^ uptake mechanisms including the low affinity constitutive transporter Trk, the 12-transmembrane domain transporter Kup, and the high affinity P-type ATPase transporter Kdp [42,57]. Defective K^+^ uptake limits bacterial growth under standard extracellular K^+^ conditions, a property used to characterize heterologously expressed ion channels by the rescue of cell growth [43]. TK2463 bacteria were transformed with *hAQP1* and control plasmids; protein expression was driven by the IPTG-inducible tac-promoter [43]. In medium with 4 mM KCl, expression of wild-type hAQP1 improved growth 0.3 to 0.4-fold over levels seen in hAQP1-transfected cells not IPTG-induced (Supplementary Figure S4), as assessed by quantification of growth curve slope values (from 6 to 30 h; Supp Fig S4.B) and as area under curve values (Supplementary Figure S4C). With hAQP1 expression, *E. coli* growth was approximately doubled as compared with IPTG-treated empty vector control cells. To test for potentiation of growth, hAQP1-expressing TK2463 cells were treated with the agonist CPT-cGMP; a nonsignificant trend toward increased growth as compared with hAQP1-expressing cells without cGMP was observed (Supp. Fig. S4), suggesting baseline ion channel activity was sufficient. The demonstration that hAQP1 ion permeability can be reproduced in an independent model system, *E. coli*, illustrates feasibility of additional screening tools. Future studies might take advantage of cell-specific differences in signaling pathways to identify factors which influence gating of diverse classes of novel candidate AQP ion channels.

## Discussion

The primary outcome of this work was the demonstration that the yeast screening method is a feasible and effective approach for detecting and characterizing properties of AQP ion channels, as demonstrated for AQP1 and two other known AQP ion channels, Arabidopsis AtPIP2;1 and mammalian AQP6. AQP channel properties that have been characterized in other cell types were found to be retained in a yeast model, providing support for the method as a tool for mass screening of diverse AQP classes for potential ion channel functionality. Effects of ion channel but not water channel inhibitors in reducing yeast growth showed that rescue depended specifically on hAQP1-mediated cation permeability. New findings include insights into pH-dependent actions of the most effective AQP1 ion channel inhibitors, identification of the divalent cation Co^2+^ as a novel inhibitor of the AQP1 ion channel, AQP1 insensitivity to Mn^2+^ and Ni^2+^, and evidence that human AQP6 also functions as an ion channel. Site-directed mutations of structural and regulatory domains reflected known structure-function roles described previously [3]. Aquaporin-mediated water permeability confirmed by osmotic stress effects proved valuable for assessing integrity and plasma membrane trafficking of introduced wild-type and mutant AQP channels.

Heterologous expression systems such as yeast and bacteria are valuable tools for the biological investigation of ion channels [7,58]. Yeast transport mechanisms for K^+^ and Na^+^ [59] have been used to create platforms for ion channel discovery based on genetic deficiency for K^+^ uptake Δ*trk1*Δ*trk2* [60], or Na^+^ efflux Δ*ena1-4*, Δ*nha1* [61]. The yeast expression system has been used to investigate effects of pH on AQP water, glycerol and urea permeabilities [26,62–65]. Exploring other classes of channels, yeast systems have proven useful for deciphering structure-function relationships [66,67], validating unusual ion channels lacking signature sequence motifs [68]; or studying channels not natively expressed in plasma membrane but accessible in isolated yeast vacuoles [69,70]. The ability of the yeast expression system to detect weak or non-selective ion channels allowed characterization of mutant KAT1 channels not evident in the *Xenopus* oocyte model [71].

Yeast intracellular messenger systems respond to a broad array of established pharmacological agents [72–74], facilitating evaluations of ion channel regulatory mechanisms. Yeast intracellular messenger systems responding to known protein kinase stimulators [74,75] can be used to assess roles of post-translational modifications in controlling gating mechanisms of AQPs [12]. hAQP1 integration into the yeast plasma membrane was reduced by fusion-protein tagging, consistent with prior work showing the C-terminal domain influenced regulation by phosphorylation and ion channel activation [14]. The hAQP1 C-terminal fusion protein failed to rescue yeast growth despite minor levels of membrane localization, whereas the N-terminal construct which also was present at low levels in plasma membrane did allow rescue of yeast growth. The rescue of yeast growth was increased by agents that activate hAQP1 ion channels, and blocked by the non-selective protein kinase inhibitor H7, consistent with the proposed role of phosphorylation in modifying AQP ion channel availability [2,14].

Inhibition of hAQP1-rescued yeast growth was benchmarked to responses previously published for modulators for AQP1 ion and water channel activity. Pharmacological sensitivities of AQP1 ion conductance were replicated by the yeast system, using growth as a proxy measure of ion conductance. The rescue of growth was inhibited strongly by the selective hAQP1 ion channel blockers AqB011, 5HMF, and partially by KeenMind. AqB011 and 5HMF were the most effective in inhibiting CY162 growth. Although no significant pH dependence of the growth response with hAQP1 in CY162 cells was evident (consistent with findings reported here for voltage clamp recordings in the oocyte expression system), inhibitory effects of the two most effective AQP1 ion channel blockers AqB011 and 5HMF did show pH sensitivity, with left-shifted dose-response effects on yeast growth that indicated higher potency at neutral pH than at acidic values. Work here is the first to suggest a pH-sensitive inhibition by these agents. In prior work, we showed mutating two conserved arginine residues in loop D of hAQP1 resulted in delayed activation of the ion conductance and reduced sensitivity to block by AqB011. Substituting loop D residues with proline resulted in position-dependent effects on ion conductance amplitude, suggesting the loop D structural conformation influences hAQP1 channel gating [17]. 5HMF inhibition of AQP1 mediated ion currents in *Xenopus* oocytes was tested at neutral pH [33]. Possible pH-sensitive effects of AqB011 and 5HMF might reflect proton competition for the binding site, or conformational changes resulting from pH sensitive amino acids in regions crucial for hAQP1 ion channel function. Not surprisingly, the hAQP1 water transport agonist, AqF026 [48] showed no effects, corroborating the observation that water permeation is not a rate-limiting factor for yeast growth in CY162 cells. Acetazolamide, a non-specific agent suggested to block AQP water channels, showed a trend towards growth reduction that was not dose-dependent, and might reflect an off-target effect on a sensitive protein such as carbonic anhydrase [51]. Modest effects of KeenMind in reducing growth rescue might be attributed to the presence of the hAQP1 ion channel blocker Bacopaside-I in the plant extract mixture in addition to the major component Bacopaside-II which blocks the intrasubunit water pore whereas the Bacopaside-I compound is more effective in blocking the hAQP1 central pore ion channel [49,50]. Validating known properties of AQP1 ion channels, yeast growth responses showed K^+^ flux dependence, and sensitivities to identified pharmacological activators and inhibitors. Under hypertonic external osmotic stress conditions, the membrane expression of water permeable AQPs caused inhibition of growth by water loss, showing these wild-type and mutant AQPs were present in the membrane, and folded correctly into functional water channels. However, water channel activity was not required for the rescue of growth in K^+^-transport deficient CY162 cells.

Cation sensitivity of AQP channels has been used for functional analyses. Differential block of yeast growth rescue by divalent cations recapitulated results of previous electrophysiological studies in oocytes [11,31,32]. Divalent block of water and glycerol fluxes has been reported previously. Inorganic metals such silver and gold containing compounds inhibit water channel activity of plant [76] and human aquaporins such as hAQP3 in erythrocytes and hAQP7 in adipocytes [77,78]. Effects of Mn^2+^ or Ni^2+^ on AQP ion conductance have not been previously reported. In hAQP3, Cu^2+^ (1 mM) inhibited water and glycerol fluxes, and Ni^2+^ (1 mM) inhibited pH-sensitive water but not glycerol flux [79,80]. Using an optical lithium sensor, cation permeability of hAQP1 in CY162 was confirmed independently. An *E. coli* mutant strain defective in K^+^ uptake provided additional support for capacity to measure ion conductivity in hAQP1 using expression systems compatible with mass-screening.

Other dual water-and-ion channel AQPs also were capable of rescuing growth in CY162, as seen for AtPIP2;1 (a monovalent cation channel) and mammalian AQP6 (proposed to be an anion channel, but also shown to enable cation permeation in flux assays) [81]. Interestingly, AQP channels expressed in yeast rescued growth without applied stimuli; hAQP1 was tested without added cGMP, and AQP6 was not activated by Hg^2+^, suggesting baseline levels of AQP channel activity in the yeast system were sufficient to enable K^+^ dependent growth over the three-day assay duration. Since the probability of a channel being in the closed state is never completely zero, channels are expected to conduct some ions even in non-stimulated conditions [2]. It appears cation entry sufficient to rescue growth occurred despite the absence of activating ligands during extended assay periods. Native serine-threonine and tyrosine kinase signaling pathways are known to modulate AQPs [82]; it is possible their baseline activities in yeast might prime expressed AQP channels to activate despite low ligand levels, or alternatively endogenous levels of intracellular signals (such as cGMP for AQP1) might be sufficient without potentiation to permit growth rescue [83]. Success reported here indicates the yeast assay system is highly sensitive, picking up cation permeation at levels below threshold for detection by other methods such as voltage clamp in oocytes. This sensitivity allows first pass identification of novel candidates without requiring *a priori* knowledge of the activating pathway. It is interesting to note human AQP6 proved difficult to characterize as an ion channel in the oocyte expression model, whereas rat AQP6 showed ionic currents in response to acidification or binding of the non-physiological agent Hg^2+^ [25,81]. Data here are the first to suggest that human AQP6 also is capable of carrying ions.

AtKAT1 served as a positive control in the yeast screening studies [40,84]. The KAT1-encoded channels exhibit significant selectivity for K^+^ ions compared with other monovalent cations, and introduction of this gene has been shown to fully reinstated K^+^ uptake in yeast mutants [85]. Comparisons across different treatment conditions allowed the assessment of innate pH sensitivity in yeast. Growth at pH 5.7 and 6.4 appeared optimal for yeast growth, which was slightly reduced at higher and lower pH values but does not prevent incorporation of pH as a condition tested in screening assays when standardized against a positive control benchmark.

Limitations of the yeast high-throughput expression model for screening AQP ion channels include variation in signal peptide recognition sequences that might limit expression or intracellular targeting, interference by yeast signalling pathways resulting in false-positive phenotypes, or barriers imposed by yeast cell walls that limit access of pharmacological agents depending in part on growth stage [86–88]; for example, yeast cell walls thickened by mannoprotein layer formation in the stationary phase are stiff and less permeable than in the growth stage [89]. Another challenge is that water and glycerol permeability conferred by multi-substrate AQPs could interfere with yeast cellular homeostasis and cause nonspecific effects on phenotypes.

## Conclusion

Given that AQPs are expressed in all domains of life, an expanded understanding of their diversification in terms of function and mechanisms of regulation is likely provide unexpected insights into conditions influenced by fluid and ion homeostasis, for issues ranging from agriculture to medicine. Since the first characterization of cloned AQP1 as a water channel [90], appreciation of the broad roles served by AQP channels has accelerated. Parallel progress in drug discovery for AQPs is a compelling goal [36] for therapeutics, interventions, and tools for basic research. As pioneered here for hAQP1, outcomes of this study establish the yeast system as a valuable approach for efficient high-throughput unbiased discovery of novel AQP ion channels and pharmacological tools. Future screening of large chemical libraries for the identification of potent AQP modulators will be of keen interest.

## Supplementary Material

Supplementary Figures S1-S4 and Tables S1-S2

Supplementary Movies S1 and S2

## Data Availability

All supporting data and results are presented in the paper. Additional details are available on request from the corresponding author.

## Open Access

Open access for this article was enabled by the participation of University Of Adelaide in an all-inclusive *Read & Publish* agreement with Portland Press and the Biochemical Society under a transformative agreement with CAUL.

## Abbreviations

AQP: aquaporin
hAQP1: human AQP1
IPTG: isopropylβ-D-1-thiogalactopyranoside
PKA: protein kinase A
PKC: protein kinase C
