# Supplementary material for: Characterization of human aquaporin ion channels in a yeast expression system as a tool for novel ion channel discovery
Source: Biosci Rep. 2024 Aug 28;44(8):BSR20240542. doi: 10.1042/BSR20240542 (PMC11358751; doi:10.1042/BSR20240542)

Supplementary Information

Tables

Supplemental Table S1: Summary of yeast strains used in this study.

Supplemental Table S2. Site-directed mutations known to alter HsAQP1 ion channel properties, or predicted to impair function based on equivalent mutations in other AQPs.

Figures

Supp. Figure S1. Effect of pH on hAQP1 ionic currents in the oocyte expression system.

Supp. Figure S2. Membrane expression of hAQP1 wild type and mutant constructs assessed by susceptibility to osmotic stress.

Supp. Figure S3. Growth analysis of hAQP1 mutations affecting pH sensitivity or water and ion permeability of hAQP1.

Supp. Figure S4. Growth rescue by hAQP1 expression in K^+ transport-deficient E. coli.

Supplemental Videos

Supp Movie 1. Z-stack 3D video for GFP-hAQP1 tagged fluorescence.

Supp Movie 2. Z-stack 3D video for hAQP1-DsRed tagged fluorescence.

Supplemental Table S1: Summary of yeast strains used in this study.

Strain	Reference	Phenotype	Genotype
CY162	Ko and Gaber et al. (1991) [91]	K^+ sensitive	MATα ura3–52 trk1Δ his3Δ200 his4–15, and trk2Δ:: pck64
aqy1aqy2	Leitão et al. (2014). [30]	Osmotic stress sensitive	Mat α; leu2::hisG; trp1::hisG, his3::hisG; ura352 aqy1D::KanMX aqy2D::KanMX

Supplemental Table S2. Site-directed mutations known to alter HsAQP1 ion channel properties, or predicted to impair function based on equivalent mutations in other AQPs.

hAQP1 mutation	Structural location	Effect(s) on channel function (as compared to wild type)	References
R159A+R160A	loop D gate	Slowed response to cGMP and impaired ion conductance in HsAQP1 R159A+R160A (reduced by ~60-80%).<br>Normal water channel activity.	Kourghi et al., 2018. [17]<br><br>Yu et al., 2006. [18]
D158P	loop D gate	Significant block of hAQP1 ion channel activation (reduced to ~10%).<br>Normal water channel activity.	
R160P	loop D gate	Decreased ion conductance of hAQP1 (to ~30%).<br>Normal water channel activity.	
G166P	loop D gate	Enhanced amplitude conductance response after cGMP stimulation (~4-fold increase).<br>Normal water channel activity.	
E17N	TM1	Knockout of AQP ion channel function in *Drosophila* BigBrain (Dm BIB E71N). ICC confirmation of plasma membrane expression.<br>Loss of water and reduction of ion channel activity by an equivalent mutation in AQP1 (Hs AQP1 E17N).	Yanochko and Yool, 2002. [56]<br><br>Yool. 2007. [92]
G57N	TM2	Decreased water flux in RnAQP1 G57N (to ~10% of wild type), attributed to failed membrane targeting of channels.	Liu et al., 2005. [55]
G72W	water pore	Knockout of AQP ion channel function in *Arabidopsis* AtPIP2;1 G103W. Partial water channel activity retained in a coexpression assay.	Byrt et al., 2017. [11]

TM = transmembrane domain; Hs = *Homo sapiens*; Rn = *Rattus norvegicus*; Dm = *Drosophila melanogaster*. ICC = immunocytochemistry

Supplemental Figure S1. Effect of pH on hAQP1 ionic currents in the oocyte expression system. (A) Current-voltage relationships compiled as mean ± SEM recorded by two-electrode voltage-clamp from oocytes expressing hAQP1 channels, in recording salines at pH 5.1, 6.1 and 7.4, before (initial, blue) and after activation by 20 μM CPT-cGMP (red), and after block by 0.6 mM $CdCl_2$ (green). **(B)** Currents recorded from representative hAQP1-expressing (left side) and non-AQP1-expressing control oocytes (right side) at voltage steps (from −120 to +60 mV, in 10 mV intervals) from a holding potential of −40 mV. (**C**) Histogram summary of compiled whole cell conductance values, calculated from linear fits of current-voltage plots for hAQP1 expressing (left) and non-AQP1 control (right) oocytes before (init) and after stimulation by CPT-cGMP (+cGMP). Subsequent effects of Cd^{2+} application (+$CdCl_2$) was tested only for AQP1-expressing oocytes. Histogram data show results from two independent batches of oocyte preparation, 2 oocytes each; with error bars showing mean ± SD. Statistically significant differences determined by ANOVA with post-hoc Bonferroni tests are shown as *p < 0.05 or **p < 0.01; ***p < 0.001; or ns (not significant).

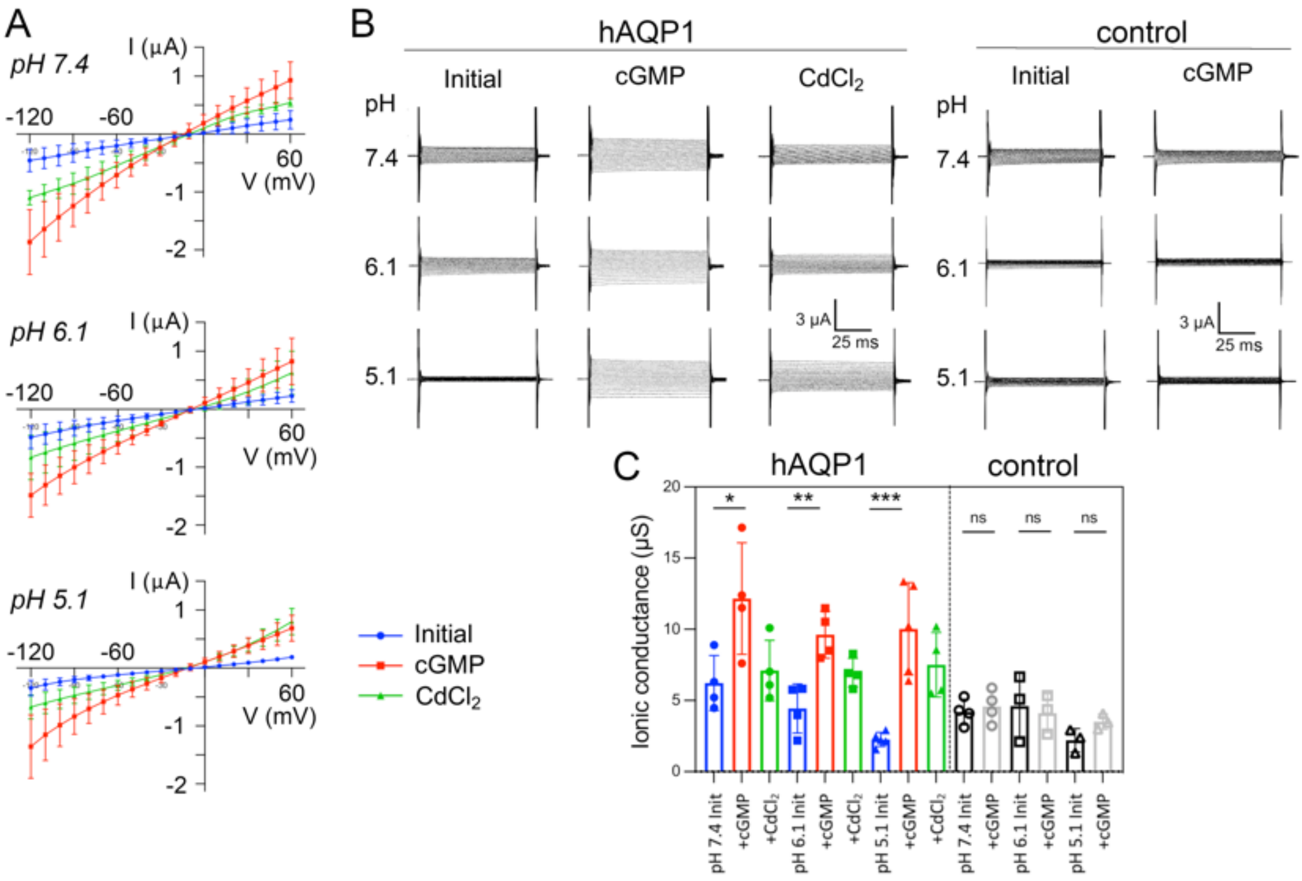

Supplemental Figure S2. Membrane expression of hAQP1 wild type and mutant constructs assessed by susceptibility to osmotic stress. **(A)** Growth of *aqy-null* cells transformed with hAQP1 wild-type or hAQP1 mutant channels including the double mutant R159A+R160A (indicated as "RRAA"), single site mutations D158P, R160P, G166P, G57N, G72W, or E17N, or with the fusion proteins hAQP1-DsRed or GFP-hAQP1, as compared to empty vector transfected cells (colors as indicated in the key). AUC plots (insets) show differential osmotic sensitivities for the different constructs in hypertonic K^+ and high Na^+ media. YNB alone or with osmotically equivalent sorbitol were used as control media in which cells grew equally well. AUC data are from two independent experiments each with triplicate samples; error bars show mean ± SD. Statistically significant differences determined by unpaired Students t-test are shown as *p < 0.5, and ns (not significant). (**B**) Yeast expressing hAQP1 in permissive media with 100 mM KCl showed equivalent growth across treatments, indicating block was not due to general toxicity.

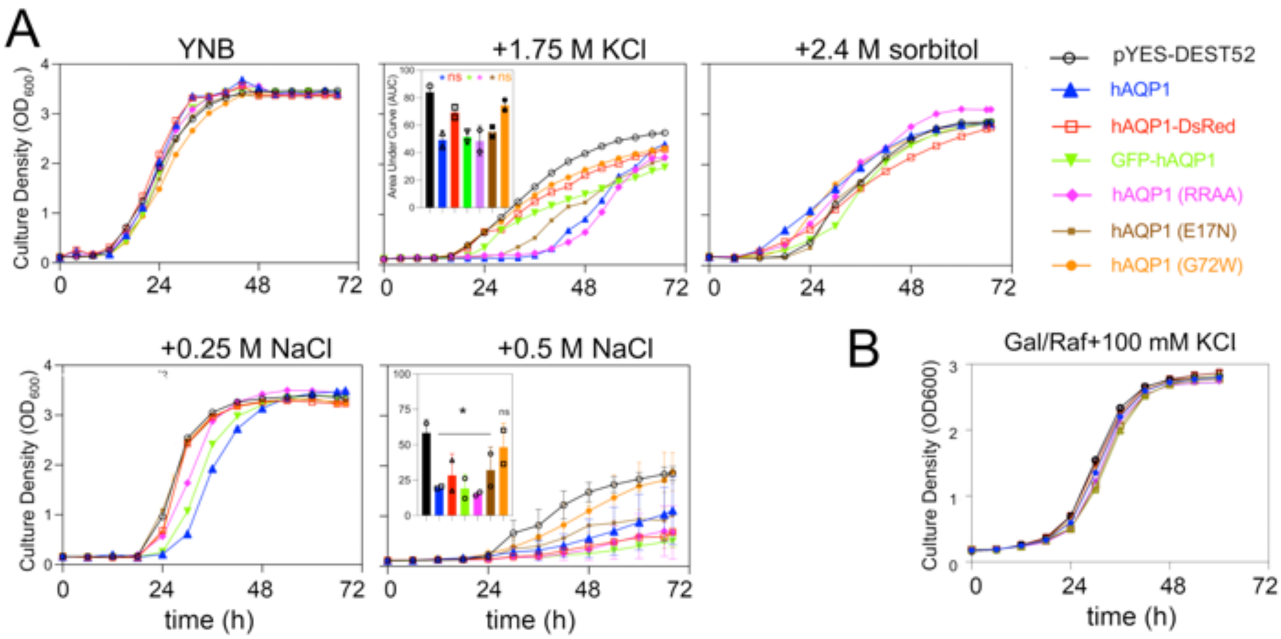

Supplemental Figure S3. Growth analysis of hAQP1 mutations affecting pH sensitivity or water and ion permeability of hAQP1. CY162 cells transformed with or hAQP1 mutant channels including the double mutant R159A+R160A (indicated as "RRAA"), single site mutations D158P, R160P, G166P, G57N, G72W, or E17N (colors as indicated in the key) were evaluated for growth rescue in 6 mM KCl at different pH values. AUC histograms show compiled data for growth rates from two independent experiments, each in triplicate, with error bars showing mean ± SD. Statistically significant differences determined by ANOVA with post-hoc Bonferroni tests and shown as *p < 0.05; **p < 0.01; ***p < 0.001; ****p < 0.0001; or ns (not significant).

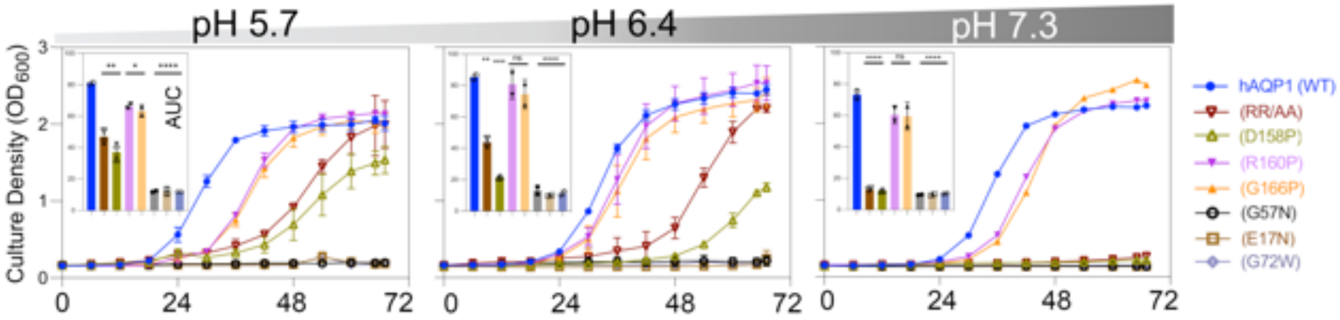

Supplemental Figure S4. Growth rescue by hAQP1 expression in K+ transport-deficient E. coli. **(A)** Growth measured by optical density (OD_{600}) in 4 mM KCl media as a function of time for K+-uptake deficient *E. coli* TK2463 cells transformed with pET-DEST42 vector (selected by 100 µg/mL carbenicillin), with or without the gene for hAQP1, with or without expression inducer (0.6 mM isopropylβ-D-1-thiogalactopyranoside, IPTG), and with and without 20 µM CPT-cGMP. Data compiled from duplicate experiments with two technical replicates each are shown as mean ± SD. **(B)** Compiled data for growth rates measured as growth curve line slopes between 6-30 hours (grey highlighted). **C)** Compiled data for AUC values. Significant differences analyzed by unpaired *t* test are reported as * p< 0.05; and ns not significant.

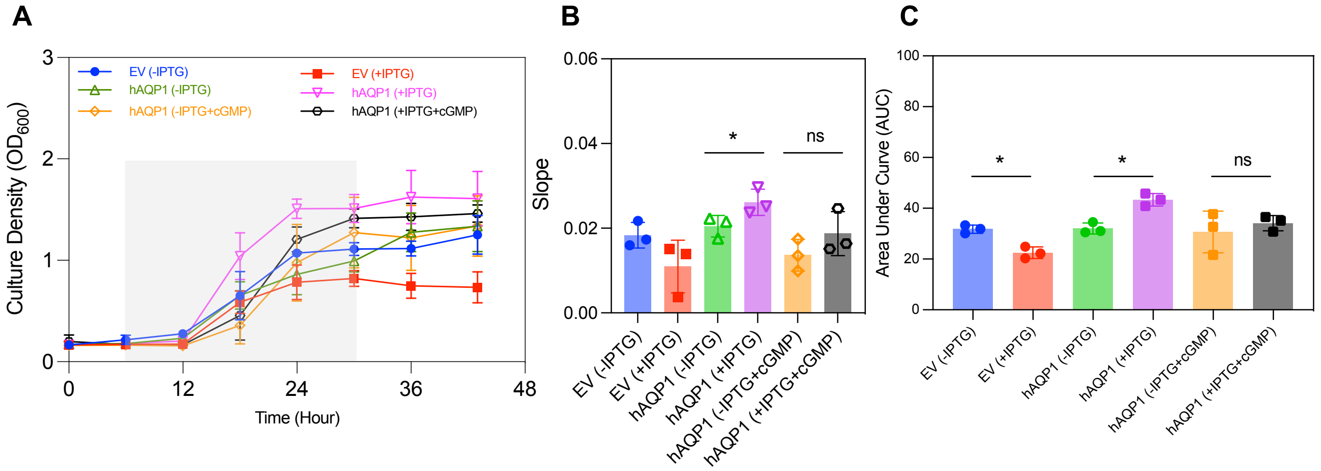

Supplement: Supplementary Figures S1-S4 and Tables S1-S2 [file BSR-2024-0542_supp.pdf]
